# The evolving schistosomiasis agenda 2007-2017—Why we are moving beyond morbidity control toward elimination of transmission

**DOI:** 10.1371/journal.pntd.0005517

**Published:** 2017-04-20

**Authors:** Charles H. King

**Affiliations:** 1 Center for Global Health and Diseases and WHO Collaborating Centre for Research and Training for Schistosomiasis Elimination, Case Western Reserve University, Cleveland, Ohio, United States of America; 2 Schistosomiasis Consortium for Operational Research and Evaluation, University of Georgia, Athens, Georgia, United States of America; University of Washington, UNITED STATES

Over the past decade at PLOS NTD, our thinking about schistosomiasis control has changed dramatically. Before going further in this article, though, it is especially important for me, as an editor, to ask everyone in the NTD world to be much more precise now about their terminology when referring to ‘schistosomiasis control’.

One can no longer talk about schistosomiasis and its control in loose fashion. Properly, schistosomiasis is the *morbidity and disease* that are caused by *Schistosoma* blood fluke *infections*, and the two states, infection and disease, should not be conflated when we are discussing control program objectives. I am not splitting hairs when I say that you absolutely cannot be ‘infected by schistosomiasis’. Schistosomiasis develops because you have been infected with *Schistosoma* parasites, and the distinction is important. Control of each entity is different, and when we discuss control in the future we should not mistake one for the other.

We now know that a program of annual or every-other-year mass drug administration [1, 2] can control morbidity (schistosomiasis) [3], but often without achieving any significant reduction in local parasite transmission (the process of *Schistosoma* infection) [4]. However, we also know that the disease schistosomiasis will be ultimately abolished when we reach the goal of total elimination of local *Schistosoma* transmission. Because many mass drug administration (MDA) programs have now attained very low levels of *Schistosoma* prevalence [5], it is notable that the World Health Assembly in 2012 called, for the first time, for *Schistosoma* transmission interruption ‘wherever possible’ (WHA 65.21).

Clearly, a standard morbidity-control program, or a more aggressive infection-control program, each has its own objectives and practices, and each has its own costs and benefits, so we should pay attention to these differences. In the past, a post-colonial worldview of the 20^th^-century World Health Organization (WHO) led to the notion that the only feasible objective for schistosomiasis-control programs was to get modest morbidity control at limited cost. Disappointingly, morbidity control by repeated treatment was the only objective to be pursued, and this focus may have sold endemic populations short. Drugs such as praziquantel and oxamniquine were too expensive for general use, so it was age-targeted treatment of high risk schoolchildren that was recommend as a ‘best practice’ at that time. Because of the risk of rapid reinfection, annual retreatment was considered by some to be ‘futile’, and non-treatment of some segments of the population (preschoolers and adults) was considered acceptable [6]. Subsequently, a very important barrier to effective implementation of preventive MDA was breached when the cost of praziquantel was reduced more than 90% through the Schistosomiasis Control Initiative’s and other programs’ engagement with generic drug manufacturers [7]. This considerably changed the calculus for control.

New, more sensitive diagnostics are teaching us that standard anti-schistosomal therapy with praziquantel is only partially effective in reducing the burden of active infection in any given round of treatment, and so MDA is likely never to be fully curative [8, 9]. Whereas control and prevention of schistosomiasis can be achieved by early and repeated treatment of active *Schistosoma* infections, local transmission usually continues, and continued residence in endemic areas carries the risk of frequent exposure to reinfection [10].

Implementation of national school- and community-based MDA programs have yielded significant benefits in Uganda, Burundi, Rwanda, Cameroon, Mali, Burkina Faso, Niger, China, Laos, and other nations. Results have included significant reductions in the prevalence of heavy infections and of many advanced forms of schistosomiasis. But with these successes, the impact of MDA was seen to plateau over time. This led project managers to ask “What next?” i.e., what more could be done to completely eliminate the impact of *Schistosoma* infection? Practical experience and dynamic modeling of MDA effects on *Schistosoma* transmission both indicate that premature interruption of MDA programs will often result in re-emergence of infection prevalence in the space of a few years [4]. Rather than mark time indefinitely using only MDA, we are searching for means to augment control, with the aim of entirely suppressing parasite transmission and ultimately lifting the need for MDA intervention. Modeling efforts are currently being used to gauge the potential costs and benefits of introduction of snail control and comprehensive water, sanitation and hygiene (WaSH) interventions in order to more significantly limit the risks for primary infection and for re-infection after therapy [11, 12].

Now, the decision to implement recommended morbidity control vs. transmission control can depend on local resources, local ecology of transmission, and finances. Disease control, and our more aspirational infection control targets, can vary across different constituencies. The WHO has now developed a ladder of control objectives, in which a country’s first goal is to reduce morbidity by reduction and then elimination of heavy infections, followed by program enhancements to then achieve transmission-interruption [13, 14].

It turns out that our standard diagnostics, i.e., quantitative screening for eggs in stool or urine, are not sufficiently sensitive to detect the many low intensity infections that are found in lower transmission areas and post-MDA populations [15]. Mapping and remapping of *Schistosoma* infection prevalence using antigen detection tools has given us a much better appreciation of the 2017 burden of infection around the world, and especially across Africa. This new appreciation of the existing burden of *Schistosoma* infections has allowed us to realize that *Schistosoma*-associated morbidity is much more than the ‘classic’ form of schistosomiasis described in textbooks. Over the last decade, new studies have documented the fact that chronic *Schistosoma* infection means significant chronic inflammation, with many consequent ‘non-specific’ or functional symptoms such as pain, fatigue, chronic diarrhea, painful urination, painful intercourse, and secondary infertility. The multi-year detrimental impact of infection on growth, nutrition, and end-organ function means there is still a large existing cohort of patients with moderate-to-advanced disease. Even post-infection, these patients will continue to suffer from illness. It is unfortunate that there are no present recommendation for ‘intensive disease management’ (IDM) for schistosomiasis, as there are for lymphatic filariasis, leprosy, and leishmaniasis [16]. Similarly, there is not yet a ‘preschool-friendly’ formulation of praziquantel that could serve to treat more of the younger children at high risk for developing growth impairment and chronic anemia of inflammation [6].

Over the last decade, practical experience in operational research about MDA implementation has yielded new knowledge about *Schistosoma* transmission potential. During this era, recognition of ‘persistent hot spots’ among treated villages within control districts indicates that we clearly need to break both snail-to-human and human-to-snail transmission in order to fully prevent new infections. Drug-based control is ‘fragile’, in that recurrent transmission can emerge quickly, resulting in a return to pre-control levels of infection prevalence when MDA is stopped. There has to be significant behavior change relative to human water use and water contact, but this ultimately requires the provision of safe and sanitary alternative water supplies, latrines, and washing facilities. Snail control can have an intermediate impact, but it is the ‘WaSH effect’ that will be required for elimination. Because new landscapes of transmission will evolve under the pressure of MDA, there is an essential need for periodic remapping with sufficient granularity to find persistent transmission hot spots, so these can be addressed with added determination.

For the next decade, these should be some of the objectives on our to-do list:

- Broader and continued implementation of preventive chemotherapy MDA at > 75% coverage
- Completion of Phase III trials and licensing of a pediatric formulation for praziquantel
- Better coordination and interfacing of the schistosomiasis control community with programs focused on early childhood development, maternal health, and advanced schistosomiasis patient management
- Morbidity outcome studies using our new, more sensitive diagnostics, specifically to accurately quantify and better understand the impacts of early childhood schistosomiasis and of genital schistosomiasis
- Continued new drug and vaccine development
- Complex intervention trials (at a sufficiently large scale) on the elimination of *Schistosoma* transmission using combined drug, snail control, behavior change, and WaSH interventions.
